# An unusual new species of *Micraspis* Chevrolat (Coleoptera: Coccinellidae) from northeastern India

**DOI:** 10.3897/BDJ.2.e4112

**Published:** 2014-11-12

**Authors:** J. Poorani

**Affiliations:** † National Bureau of Agriculturally Important Insects, PB No. 2491, HA Farm Post, Bellary Road, Hebbal, Bangalore 560024, India

**Keywords:** *Micraspis*, Coccinellidae, India

## Abstract

*Micraspis pusillus* sp. n. (Coleoptera: Coccinellidae) is described and illustrated from the northeastern region of India. It is unusual in possessing very large eye canthus and is the smallest species of the genus known from India so far.

## Introduction

The genus *Micraspis* Chevrolat (in Dejean 1836) (Coccinellidae: Coccinellinae: Coccinellini) is mainly distributed in the Oriental, Australasian, Pacific and African regions (Slipinski 2007). From India, six species are known at present (Poorani 2002) and the genus is in need of revision as many species are not well defined. Members of *Micraspis* are unusual in their feeding habits as they are both entomophagous and phytophagous and seem to prefer plant pollen to animal prey (Slipinski 2007). *Micraspis discolor* (F.) is the most common species of coccinellid in Oriental paddy fields and is abundant on rice crop, particularly during flowering and feeds on pollen (Afsana and Islam 2001, Shanker et al. 2013). During surveys in northeastern states of India, a new species of *Micraspis* was collected and is described and illustrated here.

## Materials and methods

Male and female genitalia were cleared in 10% NaOH overnight and dissected in distilled water and transferred to glycerol for studies and imaging. After examination, the genitalia were transferred to microvials and pinned beneath the respective specimens. The following measurements were made using the measurement module of a Leica M205A stereo microscope: total length, from apical margin of clypeus to apex of elytra (TL); total width, across both elytra at their widest point (TW = EW); pronotal length, from the middle of anterior margin to the base of pronotum (PL); pronotal width at its widest (PW); elytral length along suture from apex to base including scutellum (EL). Images of whole specimens and their diagnostic characters were taken using a Leica DFC 420 camera attached to a Leica M205A stereo microscope. Composite images were generated from image stacks using Combine ZP and touched up in Adobe Photoshop Elements 11.

The specimens studied are deposited in the following collections:

NBAII – National Bureau of Agriculturally Important Insects, Bangalore, India.

UASB – University of Agricultural Sciences, Bangalore, India.

## Taxon treatments

### Micraspis pusillus sp. n.

urn:lsid:zoobank.org:act:FB88EBD4-A082-4833-8AB8-EB602212AA4C

#### Materials

- **Type status:** Holotype. **Occurrence:** recordedBy: Sunil Joshi; individualCount: 1; sex: Male; **Location:** country: India; stateProvince: Sikkim; verbatimLocality: Rumtek; **Event:** samplingProtocol: hand picking; eventDate: 2014-10-31; habitat: on bamboo; **Record Level:** institutionCode: NBAII
- **Type status:** Paratype. **Occurrence:** recordedBy: C.A. Viraktamath; individualCount: 1; sex: F; **Location:** country: India; stateProvince: Meghalaya; verbatimLocality: Ri-Bhoi, ICAR RC NEH, Barapani, Lower Shillong; verbatimElevation: 1031m; verbatimLatitude: 25°14'N; verbatimLongitude: 91°55'E; **Event:** eventDate: 2013-03-26; **Record Level:** institutionCode: UASB
- **Type status:** Paratype. **Occurrence:** recordedBy: C.A. Viraktamath; individualCount: 3; sex: 1 male, 2 females; **Location:** country: India; stateProvince: Meghalaya; verbatimLocality: Ri-Bhoi, ICAR RC NEH, Barapani, Lower Shillong; verbatimElevation: 1031m; verbatimLatitude: 25°14'N; verbatimLongitude: 91°55'E; **Event:** eventDate: 2013-3-26; **Record Level:** institutionCode: NBAII, UASB
- **Type status:** Paratype. **Occurrence:** recordedBy: Yeshwanth, H.M.; individualCount: 1; sex: Female; **Location:** country: India; stateProvince: Meghalaya; verbatimLocality: Ri-Bhoi, ICAR RC NEH, Umiam; verbatimElevation: 1031m; verbatimLatitude: 25°14'N; verbatimLongitude: 91°55'E; **Event:** samplingProtocol: Light trap; eventDate: 2013-06-03; **Record Level:** institutionCode: NBAII
- **Type status:** Paratype. **Occurrence:** recordedBy: Sunil Joshi; individualCount: 2; sex: females; **Location:** country: India; stateProvince: Sikkim; **Event:** samplingProtocol: handpicking; eventDate: 2014-10-31; habitat: on bamboo; **Record Level:** institutionCode: NBAII
- **Type status:** Paratype. **Occurrence:** recordedBy: Viraktamath, C.A.; individualCount: 1; sex: Female; **Location:** country: India; stateProvince: Assam; verbatimLocality: Digboi; verbatimElevation: 200 mts; verbatimLatitude: 27°21'55.7"N; verbatimLongitude: 095°41'59.8"E; **Event:** habitat: On ridge gourd; **Record Level:** collectionID: 2014-ix-08; institutionCode: NBAII
- **Type status:** Paratype. **Occurrence:** recordedBy: Yeshwanth, H.M.; individualCount: 1; sex: Female; **Location:** country: India; stateProvince: Assam; verbatimLocality: Margherita; verbatimElevation: 163.5m; verbatimLatitude: 27°15' 47.4"N; verbatimLongitude: 095°41'59.8"E; **Event:** samplingProtocol: Sweepnet; eventDate: 2014-ix-14; **Record Level:** institutionCode: NBAII

#### Description

**Figure 3.**
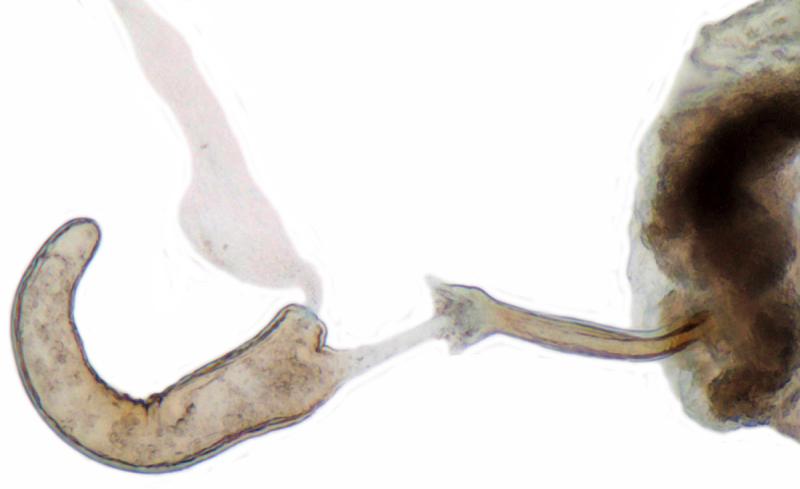
Female genitalia, spermatheca.

**Figure 4.**
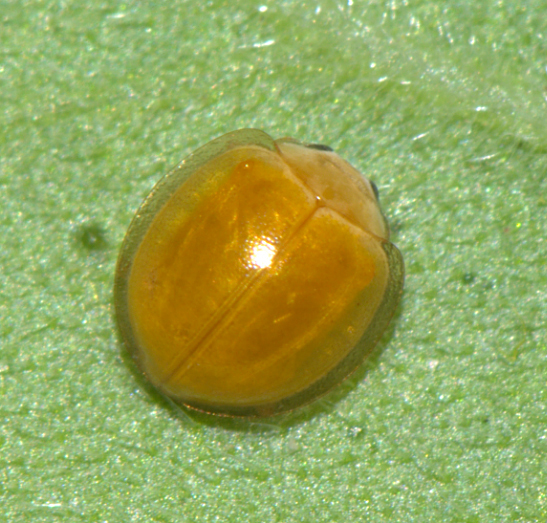
Live specimen of *Micraspis pusillus* sp. n.

TL: 3.02–3.30 mm; TW: 2.68–2.90 mm; TL/TW: 1.13–1.16; EL/EW: 0.92–0.96; PL/PW: 0.51–0.52; PW/TW: 0.55–0.57. Form (Figs 1a, b, 4), short oval to almost circular, only slightly longer than wide, moderately convex. Dorsum glabrous except head with yellowish white pubescence, more pronounced near clypeal margin. In live specimens, whole of head including ocular canthus distinctly white in both sexes; antenna yellow in female, paler in male except club distinctly darker yellow; in male anterolateral corners of pronotum slightly whitish, ventral side with hypomeron, mespimeron, mespisternum and metepisternnum with traces of white, rest of ventral side pale yellowish; in female, ventral side more or less uniform yellow. In dead specimens, head creamy yellow, pronotum and elytra uniform yellow except anterior margin of pronotum transparent; ventral side yellow except mesepimeron and mespisternum pale creamy yellow, ventral side with silvery white pubescence. Head (Figs 1c, 2a) with clypeal margin truncate between lateral projections. Eyes moderately large with a conspicuous, large canthus; inner ocular margins divergent towards posterior. Antenna 11-segmented, distinctly shorter than width of head capsule; with a three-segmented club, terminal antennomere elongate oval. Terminal maxillary palpomere securiform. Punctures on head fine and shallowly impressed, widely spaced, separated by 5-7 diameters, interspaces between punctures reticulate. Pronotum with sides rounded and narrowly upturned, anterolateral angles obtuse; anterior margin partially covering head; punctures somewhat irregular, separated by 2-5 diameters, laterally obsolete, interspaces between punctures with reticulate sculpture. Scutellum very small, triangular. Elytra laterally moderately explanate, lateral sides very narrowly margined; densely punctate, punctures larger and closer than those on pronotum, separated by 1.5-4 diameters, interspaces between punctures shiny. Prothoracic hypomera lacking foveae near anterolateral corners. Prosternal intercoxal process carinate, carinae reaching a little above middle, slightly divergent towards posterior. Anterior margin of mesoventrite medially very shallowly emarginate. Metaventrite with distinct discrimen. Legs without tibial spurs, tarsal claws appendiculate. Elytral epipleura concave, strongly descending externally, not foveolate. Abdomen with six visible ventrites, abdominal postcoxal lines (Fig. 2b) incomplete. Posterior margin of ventrite 5 truncate in female, broadly emarginate in male, ventrite 6 apically arcuate in female, truncate in male. Male genitalia (Fig. 2c, d, e, f) as shown, tegmen in inner view (Fig. 2c) with penis guide elongate, broadest anteriorly, gradually narrowed to a truncate apex, parameres in inner view slightly shorter than penis guide, apices of parameres with dense, elongate hairs, inner apical margin of paramere produced into a distinct hook-like projection, more clearly visible in lateral view (Fig. 2d); penis (Fig. 2e) with a distinct capsule, apically produced into a narrow process (Fig. 2f). Female genitalia with spermatheca (Fig. 3) as shown, infundibulum distinct, elongate tubular, anteriorly broadened, slightly narrowed towards bursa.

#### Diagnosis

*Micraspis pusillus* sp. n. can be readily differentiated from the other known Indian species of the genus by the uniform yellow body colour and unusually large eye canthus and the male genitalia also are diagnostic. The antennal insertions appear to be more dorsal than usual for Coccinellini, probably due to the large eye canthus. It is probably the smallest species of *Micraspis* in India, though other species known from this region are sometimes only slightly larger.

#### Etymology

The specific epithet is a Latin adjective in reference to the small size of this insect.

#### Distribution

India: Northeastern region (Assam; Meghalaya; Sikkim).

#### Biology

The host plants on which the specimens were collected include *Musa paradisiaca*, bamboo, and ridge gourd [*Luffa acutangula* (L.) Roxb.]. It is not known if the adults are attracted to light.

#### Taxon discussion

This species is placed in *Micraspis* by the following combination of characters given by Slipinski (2007): very small scutellum, prothoracic hypomera without foveae, tibial apices without spurs, and abdominal postcoxal lines incomplete. The general structure of male genitalia in most of the species of *Micraspis* in India and elsewhere is very similar and the penis apex shows subtle differences between species. In *M. pusillus*, the male genitalia are somewhat atypical with the penis apex being quite distinctive. The female genitalia are also unique as the shape of the infundibulum is quite different from that of other known Indian species. All the species of *Micraspis* known to the author from the northeastern region of India (*Micraspis crocea* (Mulsant), *M. univittata* (Hope) and two undescribed species) have deep but narrow eye canthus and the body colour is never uniform yellow with pronotum having infuscate to distinct black markings, scutellum and / or elytral suture blackish. *Micraspis pusillus* sp. n. is unique in having a large eye canthus and a fully white head in live specimens.

## Supplementary Material

XML Treatment for Micraspis pusillus

## Figures and Tables

**Figure 1a.**
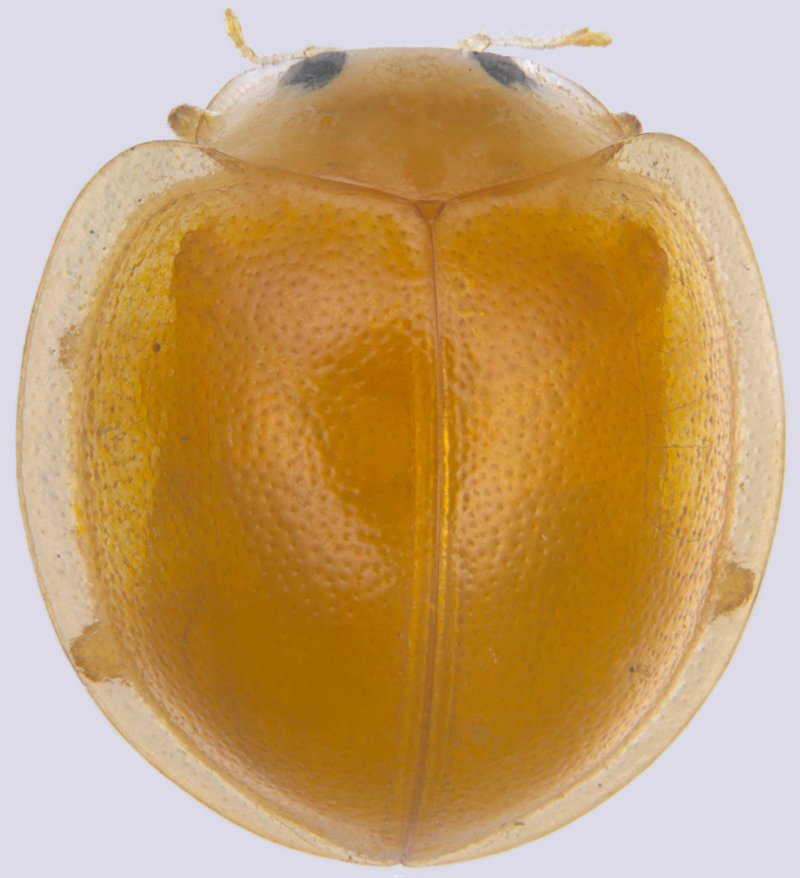
Dorsal view - live specimen

**Figure 1b.**
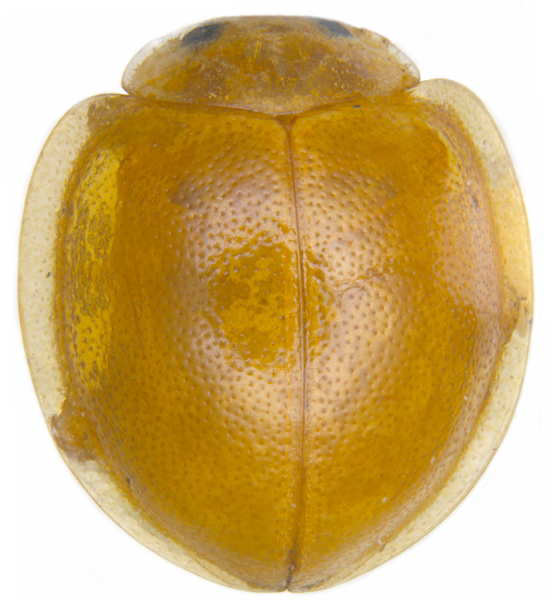
Dorsal view - dead specimen

**Figure 1c.**
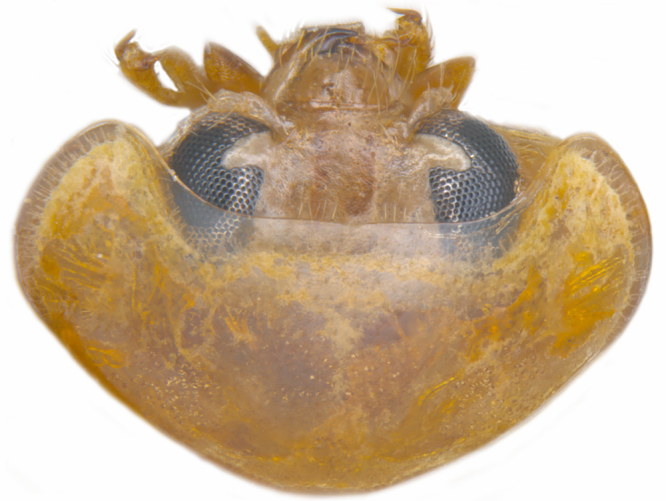
Head and pronotum, dorsal view

**Figure 1d.**
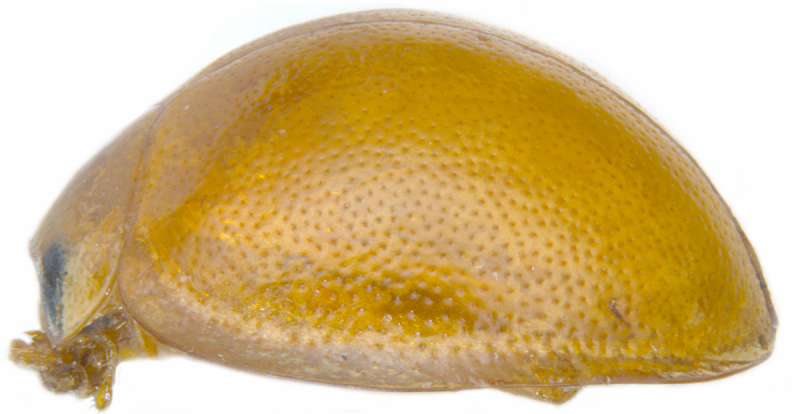
Lateral view

**Figure 2a.**
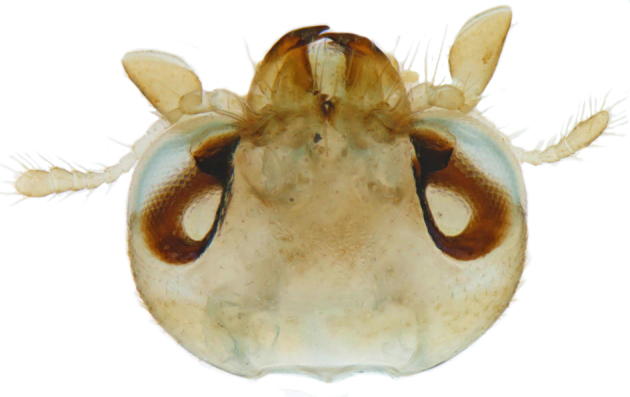
Head, dorsal view

**Figure 2b.**
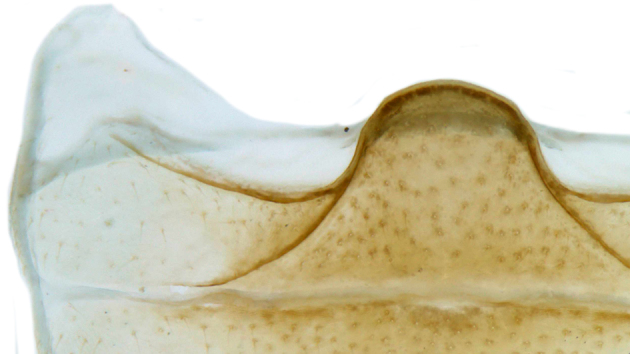
Abdominal postcoxal line

**Figure 2c.**
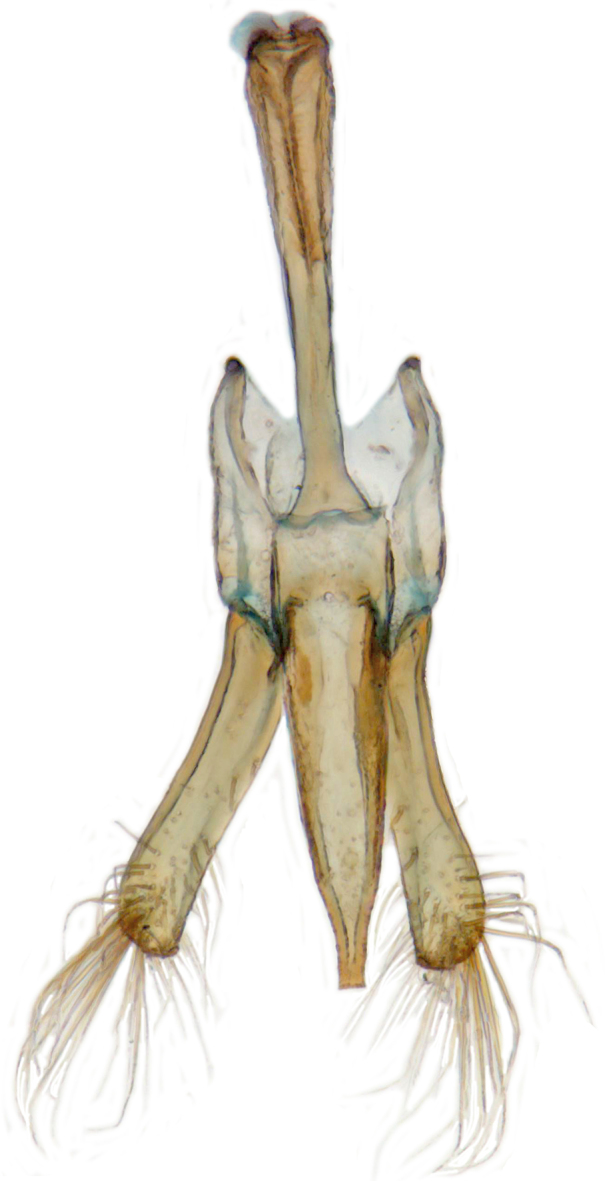
Male genitalia, tegmen, inner view

**Figure 2d.**
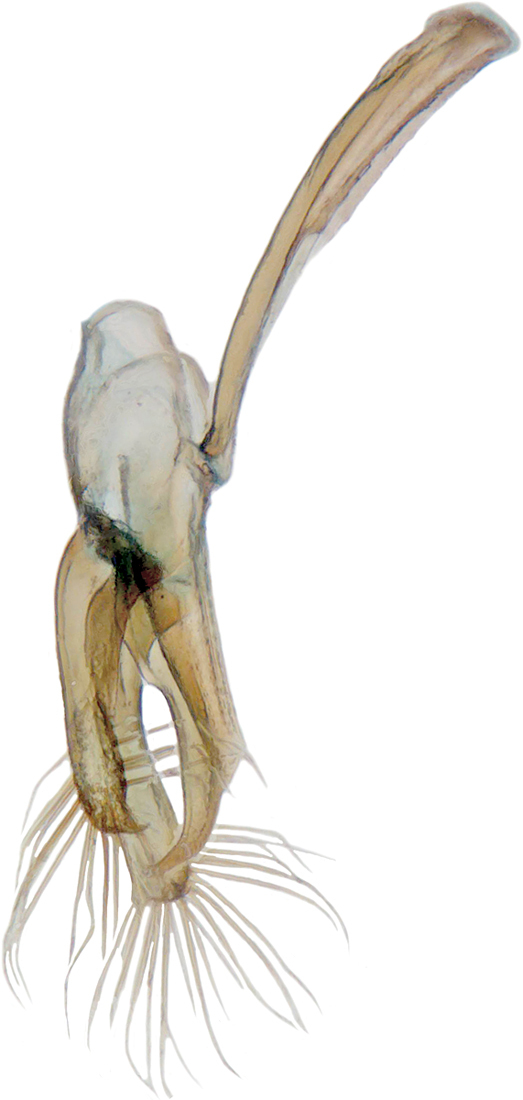
Male genitalia, tegmen, lateral view

**Figure 2e.**
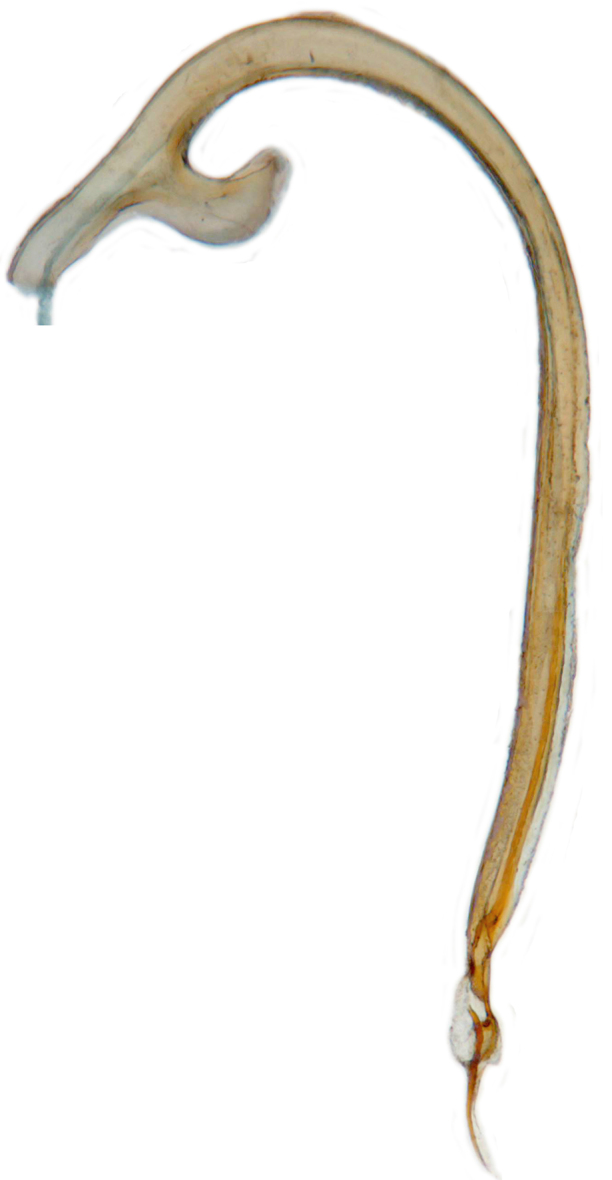
Male genitalia, penis

**Figure 2f.**
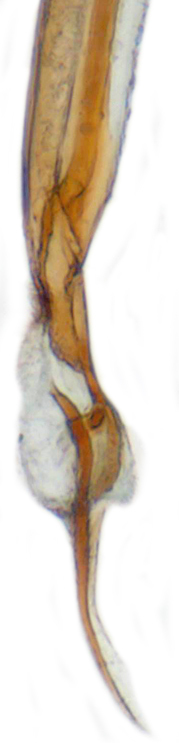
Penis apex, enlarged
